# Iron and ferritin accumulate in separate cellular locations in *Phaseolus *seeds

**DOI:** 10.1186/1471-2229-10-26

**Published:** 2010-02-11

**Authors:** Cristina Cvitanich, Wojciech J Przybyłowicz, Dorian F Urbanski, Anna M Jurkiewicz, Jolanta Mesjasz-Przybyłowicz, Matthew W Blair, Carolina Astudillo, Erik Ø Jensen, Jens Stougaard

**Affiliations:** 1 Centre for Carbohydrate Recognition and Signalling, Department of Molecular Biology, University of Aarhus, Aarhus, Denmark; 2 Materials Research Department, iThemba LABS, Somerset West, South Africa; 3 on leave from: Faculty of Physics and Applied Computer Science, AGH University of Science and Technology, Kraków, Poland; 4 International Center for Tropical Agriculture, Cali, Colombia

## Abstract

**Background:**

Iron is an important micronutrient for all living organisms. Almost 25% of the world population is affected by iron deficiency, a leading cause of anemia. In plants, iron deficiency leads to chlorosis and reduced yield. Both animals and plants may suffer from iron deficiency when their diet or environment lacks bioavailable iron. A sustainable way to reduce iron malnutrition in humans is to develop staple crops with increased content of bioavailable iron. Knowledge of where and how iron accumulates in seeds of crop plants will increase the understanding of plant iron metabolism and will assist in the production of staples with increased bioavailable iron.

**Results:**

Here we reveal the distribution of iron in seeds of three *Phaseolus *species including thirteen genotypes of *P. vulgaris*, *P. coccineus*, and *P. lunatus*. We showed that high concentrations of iron accumulate in cells surrounding the provascular tissue of *P. vulgaris *and *P. coccineus *seeds. Using the Perls' Prussian blue method, we were able to detect iron in the cytoplasm of epidermal cells, cells near the epidermis, and cells surrounding the provascular tissue. In contrast, the protein ferritin that has been suggested as the major iron storage protein in legumes was only detected in the amyloplasts of the seed embryo. Using the non-destructive micro-PIXE (Particle Induced X-ray Emission) technique we show that the tissue in the proximity of the provascular bundles holds up to 500 μg g^-1 ^of iron, depending on the genotype. In contrast to *P. vulgaris *and *P. coccineus*, we did not observe iron accumulation in the cells surrounding the provascular tissues of *P. lunatus *cotyledons. A novel iron-rich genotype, NUA35, with a high concentration of iron both in the seed coat and cotyledons was bred from a cross between an Andean and a Mesoamerican genotype.

**Conclusions:**

The presented results emphasize the importance of complementing research in model organisms with analysis in crop plants and they suggest that iron distribution criteria should be integrated into selection strategies for bean biofortification.

## Background

Iron deficiency is the most prevalent micronutrient insufficiency worldwide and the leading cause of anemia. Iron deficiency anemia and its consequences affect almost 25% of the world population (Report of the UNICEF/World Health Organization Regional Consultation, 1999). The diet in resource-poor areas consists in a few staple crops, which may provide sufficient carbohydrates but are poor in proteins and micronutrients.

Biofortified micronutrient-rich staple crops can be developed to improve human nutrition [1,2]. A target crop for biofortification is the protein-rich common bean, *Phaseolus vulgaris *[3]. A high variation in seed iron content and distribution in *P. vulgaris *genotypes has been shown, and is partly due to within-gene pool and between-gene pool differences [4].

Breeding new bean varieties can be facilitated by the use of molecular markers linked to high nutritional values [5]. Establishing which genes are important for iron uptake, its accumulation in seeds, and its bioavailability, will assist in the design of molecular markers for genes that are responsible for the high iron trait.

Iron overload is toxic for plants, while iron deficiency leads to chlorosis, reduced growth, and eventually death. Plants have therefore developed mechanisms to tightly regulate iron metabolism. The responses of non-graminaceous plants to iron deficiency include the induction of ferric chelate reductases, an increase in rhizosphere acidification, and the upregulation of ZIP type transporters responsible for iron uptake by the roots (reviewed by [6-8]). These iron deficiency responses are regulated by transcription factors of the basic helix-loop-helix family, including FIT1 in *Arabidopsis thaliana *[9-11].

Once within the plant, iron is chelated improving its mobility and protecting cells from harmful reactive oxygen species created by ferrous-iron-catalyzed Fenton reactions. Nicotianamine (NA), citrate, and the iron transport protein (ITP) are responsible for binding iron in the xylem and phloem [12-14], while ferritins were suggested to store iron in legume seeds and to provide an available iron pool in leaves [15-19].

Recent findings indicate that vacuoles are important for seed iron storage. The vacuolar iron transporters, NRAMP3 and NRAMP4, are important for the mobilization of iron during the germination of *A. thaliana *seeds [20]. Furthermore, the vacuolar transporter VIT1 is important for the distribution of iron within *A. thaliana *seeds [21]. In spite of their effect on iron distribution and seed germination, loss-of-function mutation of these genes does not affect seed iron content. In contrast, the importance of NA for seed iron homeostasis was shown by mutations in NA synthase genes in *A. thaliana *[22]. Reduction in NA synthase activity did reduce the concentration of iron in seeds.

In addition to significant variations in total iron content, even within the same species, it was shown that different legume genotypes accumulate a different proportion of the total seed iron in the seed coat, embryonic axis, and cotyledonary tissues respectively [4,23-25]. This variation in total iron and iron localization must be explained and ultimately used to advantage in biofortification efforts in beans. Therefore specific tissues important to iron storage in seeds must be identified and their iron loading mechanisms revealed.

In this study we identify the cellular localization of iron in mature seeds of three *Phaseolus *species including 13 genotypes. We have analyzed the iron localization in mature seeds of *P. vulgaris*, *P. coccineus *(runner bean), and *P. lunatus *(butter bean). We found that large concentrations of iron are accumulated in the cytoplasm of cells surrounding the provascular bundles, especially in iron-rich genotypes of *P. vulgaris *and *P. coccineus *seeds, but not in *P. lunatus*. Furthermore, we detected ferritin in the amyloplasts throughout the embryo.

## Results

### Iron accumulates in defined regions of the cotyledons of mature *P. vulgaris *and *P. coccineus *seeds

We used ICP-AES to measure the iron concentration in the seed coat, cotyledon, and embryonic axis of beans from *P. coccineus *and from seven *P. vulgaris *genotypes (Table 1). The different tissues were dissected from mature dry beans. Our results show that the iron concentrations in the cotyledons ranged from 43 to 80 μg g^-1^, and in the embryonic axis from 46 to 103 μg g^-1^. The largest variation was observed in the seed coats, in which the iron concentration ranged from 17 to 132 μg g^-1^, depending on the genotype. This corresponds to 2 to 18% of the total seed iron. Mesoamerican *P. vulgaris *genotypes G14519, G4825, DOR364, and the genotype NUA35 which is derived from a cross between CAL96 and G14519 (CIAT, unpublished), have more iron in their seed coats than the Andean *P. vulgaris *genotypes CAL96, G19833, and G21242. *P. coccineus *cotyledons and seed coats had relatively low concentrations of iron at 45 and 35 μg g^-1 ^respectively (Table 1) while G14519, a brown-seeded genotype selected from the CIAT core germplasm collection for its high-seed-iron content, had at 132 μg g^-1 ^the highest concentration of seed coat iron, and the third highest concentration of cotyledon iron at 65 μg g^-1^. The concentrations of iron in the cotyledons and seed coats of G4825, a cream and brown mottled genotype, were closest to the average of all the analyzed samples, 56 μg g^-1 ^in both cases.

**Table 1:** Iron concentration in tissues of *P. vulgaris *(*Pv*) and *P. coccineus *seeds

	**Iron concentration (μg g**^-1^**)**			Iron fraction (%)			Total iron	
**Genotype**	**Coty-ledons**	**Axis**	**Seed coats**	**Coty-ledons**	**Axis**	**Seed coats**	**μg g**^-1^	**Gene pool**

***Pv *G4825**	56 (sd 9)^a^	67 (± 3)	56 (sd 6)^a^	91	2	7	57	M

***Pv *DOR364**	43(± 3)	46 (± 1)	71 (± 5)	84	1	15	45	M

***Pv *G14519**	65 (sd 2)^a^	70 (± 3)	132 (sd 16)^b^	80	2	18	71	M

***Pv *NUA35**	80 (± 4)	103 (± 2)	61 (± 3)	90	1	8	78	N/A

***Pv *CAL96**	57 (± 2)	81 (± 6)	17 (± 2)	96	1	2	54	A

***Pv *G19833**	58 (± 2)	85 (± 4)	30 (± 2)	92	2	5	55	A

***Pv *G21242**	70 (± 9)	97 (± 5)	42 (± 2)	92	2	6	67	A

***P. coccineus***	45 (sd 5)^a^	84(± 10)	35 (sd 3)^a^	92	1	7	44	N/A

The iron concentrations in different tissues from mature seeds were obtained by ICP-AES. The iron fraction is the percentage of the total seed iron. The total iron concentration (Total iron) is based on the measured concentrations in the individual tissues. The genotypes with high seed coat iron are from the Mesoamerican (M) gene pool, while the genotypes with low seed coat iron are from Andean (A) gene pools. All the *P. vulgaris *genotypes were grown in one season in Darien, Colombia. *P. coccineus *purchased at a market in Denmark shown in Fig. 5 column 1. For some genotypes two^a ^or three^b ^individual pools of tissue were measured. N/A: non applicable. Sd: standard deviation.

**Figure 1:**
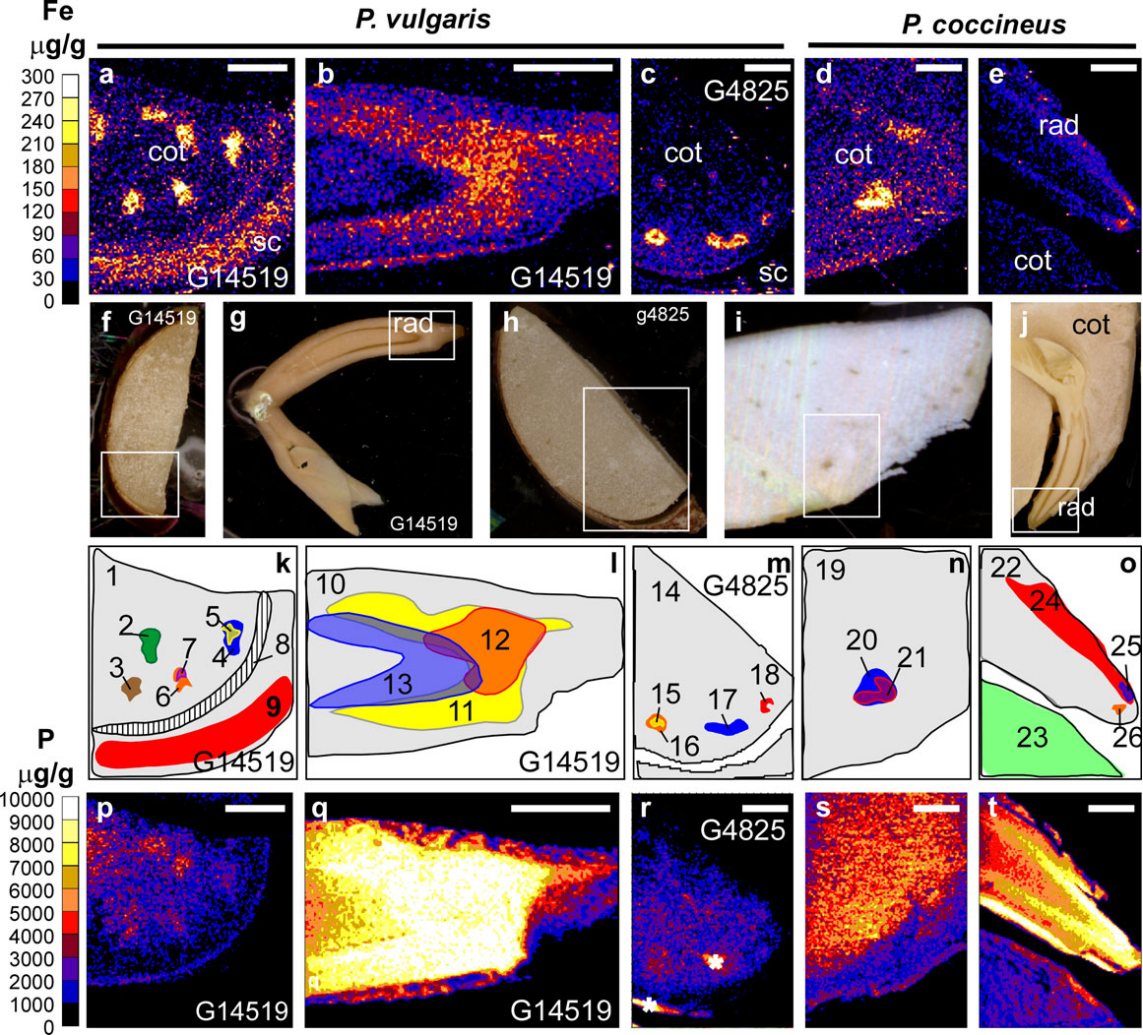
**Elemental analysis of mature seeds from *P. vulgaris *and *P. coccineus *using micro-PIXE**. Elemental maps obtained by *Dynamic Analysis *method. Maps of iron (**a-e**) and phosphorus (**p-t**). Colour scales showing concentrations are on the left side. **f **to **j **show the analyzed tissue, and the scanned area is marked with an open square. The maps **a**,**b**,**p, **and **q **represent the iron (Fe) and phosphorus (P) distribution in the cotyledon (cot) and radicle (rad) of *P. vulgaris *accession G14519 shown in **f **and **g**, respectively. The scanning of a region (shown in **h**) of the cotyledon of the *P. vulgaris *genotype G4825 is represented in the maps **c **and **r**. The analysis of the cotyledon and radicle of *P. coccineus *shown in **i **and **j **resulted in the maps **d**,**e**,**s**, and **t**. The maps **k **to **o **show the regions used for the quantification of elements displayed in Table 2. Each region has been assigned a unique number. Seed coat: sc. The * is used to mark an area that has been contaminated, and therefore elements cannot be quantified in the region. Scale bars: 0.5 mm.

We used micro-PIXE (Particle Induced X-ray Emission) to investigate the iron distribution within the individual tissues of seeds from *P. coccineus *and from the two *P. vulgaris *genotypes, G14519 and G4825 (Fig. 1). This non-destructive technique can detect and quantify iron, independent of its speciation, present at the surface of the tissue and in the near-surface layer. In comparison to chemical iron staining, the method does not require incubation in liquid solution prior to the analysis, reducing the probability of element redistribution or leaching.

**Table 2:** Average concentrations of iron and phosphorus in selected regions

K									
**Region**	**1**	**2**	**3**	**4**	**5**	**6**	**7**	**8**	**9**

**Fe (μg g**^-1^**)**	71 ± 4	60 ± 3	283 ± 21	254 ± 10	411 ± 22	341 ± 20	502 ± 46	80 ± 2	124 ± 5
Det. Lim.	(0.6)	(2)	(5.3)	(4.2)	(9.4)	(6.3)	(21)	(1.7)	(2.7)

**P (μg g**^-1^**)**	1170	1080	2490	1900	2390	2400	2720	472	33
	± 57	± 63	± 180	± 120	± 190	± 220	± 210	± 26	± 13
Det. Lim.	(10)	(41)	(135)	(103)	(186)	(155)	(288)	(26)	(19)

	**L**				**M**				

**Region**	**10**	**11**	**12**	**13**	**14**	**15**	**16**	**17**	**18**

**Fe (μg g**^-1^**)**	83 ± 5	125 ± 8	146 ± 6	68 ± 3	49 ± 5	407 ± 22	362 ± 13	257 ± 10	183 ± 11
Det. Lim.	(0.5)	(0.9)	(1.4)	(1)	(0.5)	(7.7)	(5.9)	(4.4)	(6.4)

**P (μg g**^-1^**)**	7480	10400	10600	9980	2310	4340	4330	3240	2900
	± 200	± 280	± 120	± 120	± 66	± 230	± 180	± 81	± 100
Det. Lim.	(8.7)	(17)	(26)	(21)	(8.4)	(158)	(90)	(66)	(129)

	**N**				**O**				

**Region**	**19**	**20**	**21**	**22**	**23**	**24**	**25**	**26**	

**Fe (μg g**^-1^**)**	52 ± 3	232 ± 9	297 ± 14	54 ± 3	35 ± 4	85 ± 4	187 ± 11	192 ± 15	
Det. Lim.	(0.8)	(4.2)	(5.4)	(0.4)	(0.7)	(1.1)	(4.9)	(7.3)	

**P (μg g**^-1^**)**	3950	5440	5030	6220	3100	6860	6990	9780	
	± 40	± 270	± 230	± 340	± 210	± 190	± 340	± 51	
Det. Lim.	(11)	(80)	(107)	(8.2)	(15)	(22)	(104)	(161)	

Concentration of iron (Fe) and phosphorus (P) in the regions indicated in Fig. 1k-o. The concentrations were measured using micro-PIXE. The region numbers are indicated in the table. Minimum detection limits (99% confidence level) are shown in parentheses (Det. Lim.).

Micro-PIXE analysis of cotyledons revealed that specific regions within this tissue accumulated high concentrations of iron (Fig. 1a,c, and 1d). Regions of the analyzed tissue were selected for quantification and these are highlighted in the color-coded maps (Fig. 1k to 1o). Iron-rich regions accumulated between 200 and 500 μg g^-1 ^of iron, depending on the genotype (Table 2 regions 3 to 7, 15 to 18, and 20 to 21). Differences in the concentration of iron in these regions within individual cotyledons were observed. For example the regions 3, 4, and 6, which have similar sizes, contained 283, 254, and 341 μg g^-1 ^of iron respectively. Furthermore, sub-regions with higher concentrations of iron, 400 to 500 μg g^-1 ^(regions 5 and 7), could be delineated within the regions 4 and 6.

Our analyses also show that iron is evenly distributed in the seed coat of the genotype G14519 (Fig. 1a). The average iron concentration in the seed coat region 9 was 124 μg g^-1 ^(Table 2). This value is similar to the 132 μg g^-1 ^measured for this tissue using ICP-AES (Table 1). In the embryonic axis, iron accumulation was observed near the radicle meristem (Fig. 1b and 1e). In particular, high concentrations of iron, 180 to 190 μg g^-1^, accumulated at the tip of the radicle of *P. coccineus *(Table 2 regions 25 to 26). These concentrations are higher than the 54 μg g^-1 ^average iron concentration of the analyzed radicle tissue (region 22). The radicle of G14519 also contained a higher concentration of iron near the meristematic tissue with an average of 146 μg g^-1 ^(region 12).

### Differences in iron distribution between *P. vulgaris *genotypes were detected using the Perl's Prussian blue (PPB) method

We used the PPB method to reveal additional details about the iron distribution within seed tissues. To address the efficiency of the PPB method, seeds from the same pool of beans used for ICP-AES analysis (Table 1) were soaked for 18 hours at room temperature prior to staining for iron.

**Figure 2:**
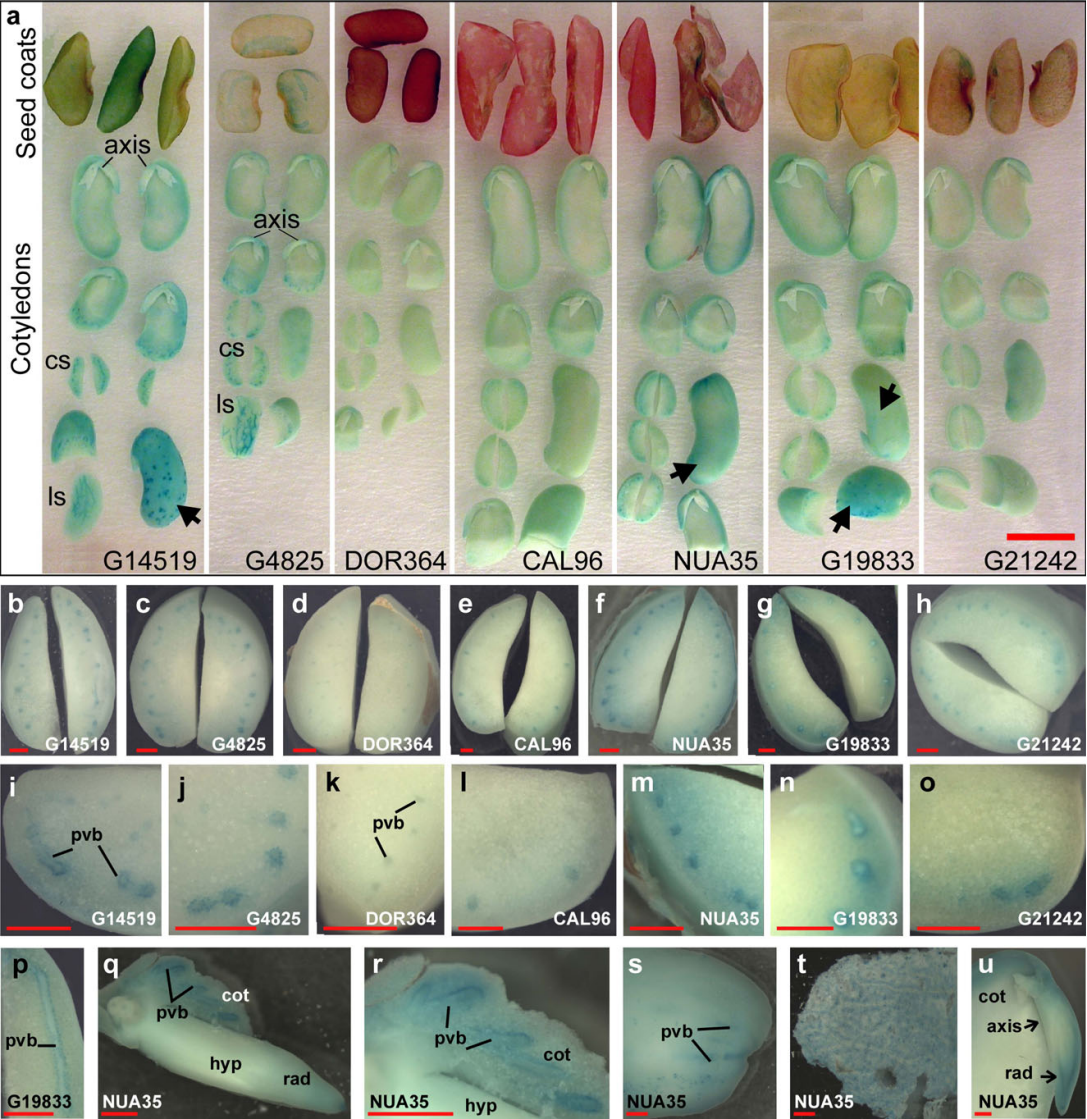
**Distribution of iron in seeds from different *P. vulgaris *genotypes**. Perls' Prussian blue (PPB) staining of *P. vulgaris *seeds that were soaked for 18 hours. **a: **The picture illustrates the variation in the stain intensity among seed coats, cotyledons, and embryonic axis (axis) of the different genotypes. Stained cross sections (cs) of cotyledons as well as longitudinal sections (ls) are shown. The outer layers of the cotyledons, proximal to the seed coats, are indicated by arrows. **b to u: **Stereomicroscopy of tissue from **a**. **b **to **h**: Cross sections of cotyledons. **i **to **o**: close-up pictures of **b **to **h **near the pro-vascular bundles (pvb). **p**: longitudinal section of pvb. **q**: hypocotyl (hyp) and radicle (rad) and attached cotyledon tissue (cot) showing pvb. **r**: close-up of the provascular tissue shown in **q**. **s**: cotyledon sample where a portion of the epidermis and proximal cell layers was removed prior staining making the pvb accessible for the Perls' solution. **t**: endosperm layer. **u**: longitudinal section of bean embryonic axis showing PPB stain of the radicle provascular and meristematic tissue. Scale bars in **a**: 1 cm and **b **to **u: **1 mm.

In agreement with the ICP-AES and micro-PIXE analysis, the genotype G14519 showed intense blue staining in the seed coats (Fig. 2a compared to Table 1 and Fig. 1). The PPB method also shows the presence of seed-to-seed variations with respect to seed coat iron content in this genotype (Fig. 2a). A unique distribution of iron was observed in the seed coats of genotype G4825. Blue staining, indicating the presence of high iron concentration, was observed in the darker brown pigmented areas of the seed coat, suggesting an association with tannins located there. In genotype CAL96 which contained only 17 μg g^-1 ^of iron in the seed coats, only small spots of blue stain could be detected near the seed hilum. G19833 and G21242, which are both mottled beans, also had light staining near the hilum. These results indicate that there is a positive correlation between iron concentration in seed coat tissues and the blue stain achieved by the PPB technique. However, the method cannot be used to detect iron in darkly pigmented seed coats, as in the case of DOR364.

The PPB method also detected high iron concentrations in the cotyledonary regions (Fig. 2), a feature previously revealed using micro-PIXE analysis (Fig. 1). Most genotypes, with the exception of DOR364, accumulated iron near the provascular tissue (Fig. 2). In G19833 iron accumulation was evident in the cells surrounding the provascular bundles (Fig. 2n and 2p). Some genotypes, in particular G14519, NUA35, and G19833 showed significant iron staining in the region near the cotyledon epidermis (Fig. 2, arrows).

Stains of the embryonic axis indicated the presence of higher iron accumulation near the radicle tip (Fig. 2q and 2u). These results correspond to the observations from the micro-PIXE analysis (Fig. 1). Strong blue stain was observed in the endosperm layer of some genotypes, as for example in NUA35, which is a high-seed-iron genotype from the Andean gene pool bred at CIAT through a backcross with G14519 (Fig. 2t). This is in agreement with ICP-AES analysis, which showed that the endosperm layer contained 270 ± 2 μg g^-1 ^and 120 ± 2 μg g^-1 ^of iron in the related genotypes NUA35 and G14519, respectively. NUA35 was also high in cotyledonary iron and had the highest overall level of seed iron.

In short, there is good agreement between the observations using micro-PIXE analysis and the PPB method. The PPB method is ideal for the detection of seed-to-seed variations, and to determine the sites of iron accumulation in a cost-effective way.

**Figure 3:**
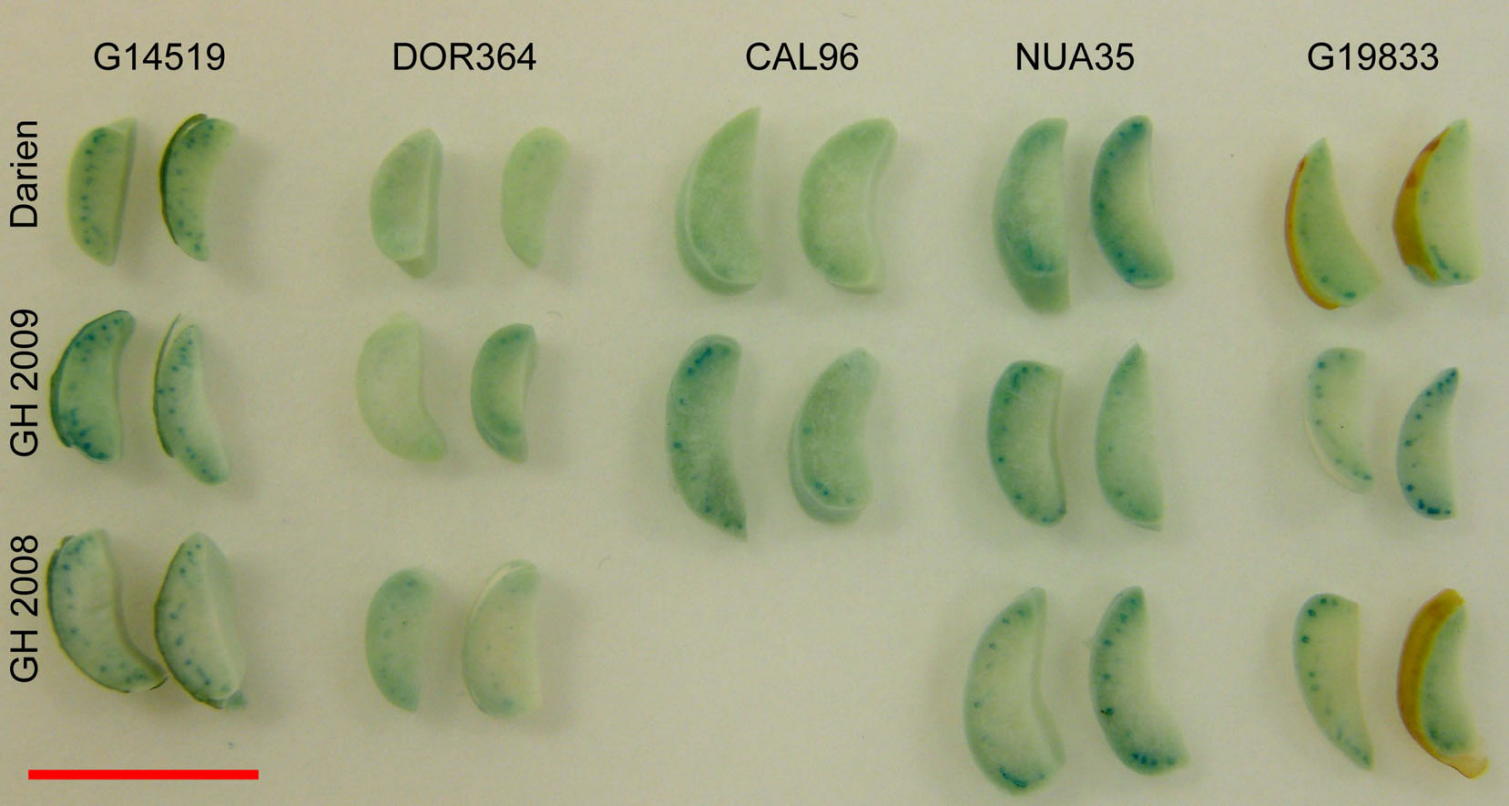
**Effects of different growth conditions on seed iron distribution**. Perls' Prussian blue (PPB) staining of seeds from five *P. vulgaris *genotypes grown in Darien, Colombia (Darien), or in a greenhouse in Denmark in 2008 (GH2008) or in 2009 (GH2009). The dry seeds were dissected and stained for 35 minutes using the PPB method. The genotypes names are shown. The scale bar is 1 cm

To address whether different growth conditions affected the iron distribution in *P. vulgaris *genotypes, we performed PPB staining on seeds from plants grown in Darien, Colombia, and on seeds from plants grown in a greenhouse (Fig. 3). The PPB staining indicate that the relative iron accumulation in the studied genotypes is stable in the used growth conditions, for example DOR364 showed the least staining in all the three environments while G14519 and NUA35 showed the most intense blue staining. Only one example of genotype × environmental effects was observed. CAL96 accumulated iron near the provascular bundles when grown in the greenhouse but not in the field. Seed to seed variations were observed occasionally, even between seeds from a single harvest from an individual plant. For example most of the seeds from the genotype DOR364 grown in the greenhouse in 2009 showed very weak blue staining, while a few showed a more intense blue color (Fig. 3, GH2009).

### High concentration of iron accumulates in the first layer of cells that surround the provascular bundles of the mature embryos of *P. vulgaris*

**Figure 4:**
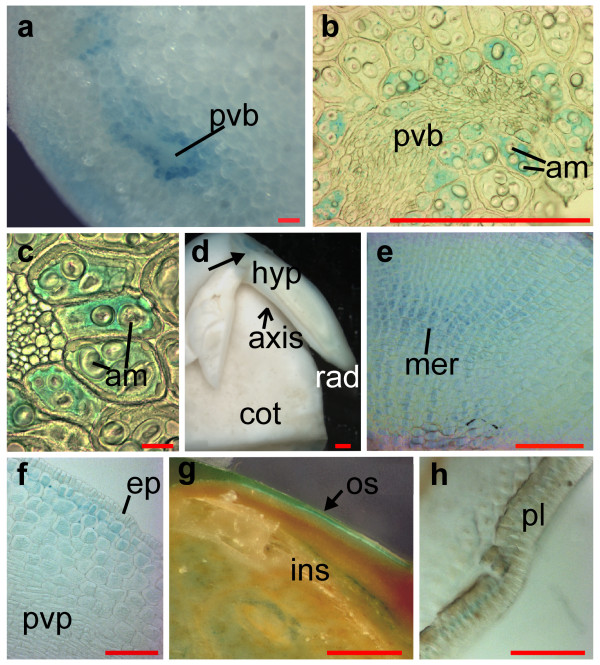
**Cellular localization of iron in the *P. vulgaris *genotype G14519**. Beans soaked for 18 hours (**a **and **e **to **h) **or unsoaked (**b **to **d**) stained with Perls' Prussian blue reagent. **a**,**d**,**g**: Stereomicroscopy. **a**: Cotyledon tissue, close-up of provascular bundle (pvb). **d**: Longitudinal section of embryonic axis (axis) including cotyledon tissue (cot), and the axis attachment zone indicated by a filled arrow. **g**: Seed coat, showing the inside (ins) part toward the cotyledons and the stain at the outer side (os) of this tissue. **b, c, f** and **h**: light microscopy of thin sections of PPB stained tissue. **b **and **c**: Close-up of cotyledon pvb, surrounded by blue stained bundle-sheath-like cells which contain amyloplasts (am). **e**: Close-up of radicle meristem showing the stained meristematic cells (mer). **f**: Section of hypocotyl tissue showing iron accumulation in the cells proximal to the epidermis (ep). **h**: Seed coat section illustrating blue stain in the palisade layer (pl). Scale bars in **d **and **g: **1 mm, in **a, b, e, f, **and **h: **0.1 mm, and in **c: **0.01 mm.

The cellular localization of iron was studied using the PPB method. Microscopical analysis of tissue sections allowed us to verify that cells surrounding the provascular bundles are responsible for iron storage in the cotyledons of the G14519 genotype (Fig. 4). In addition, the iron-specific staining is limited to the cytoplasm of these cells, while no staining was observed in the amyloplasts (Fig. 4b and 4c). Microscopical analysis confirmed that the meristematic tissue of the radicle is also rich in iron (Fig. 4e). In addition, detectable levels of iron were observed in cells near the radicle epidermis (Fig. 4f), and in the palisade layer of the seed coat (Fig. 4g). Due to the smaller cell size, the subcellular localization of iron in the radicle and palisade could not be determined in these sections.

### Cells of the provascular region are responsible for iron storage in *P. coccineus*, but not in *P. lunatus *beans

**Figure 5:**
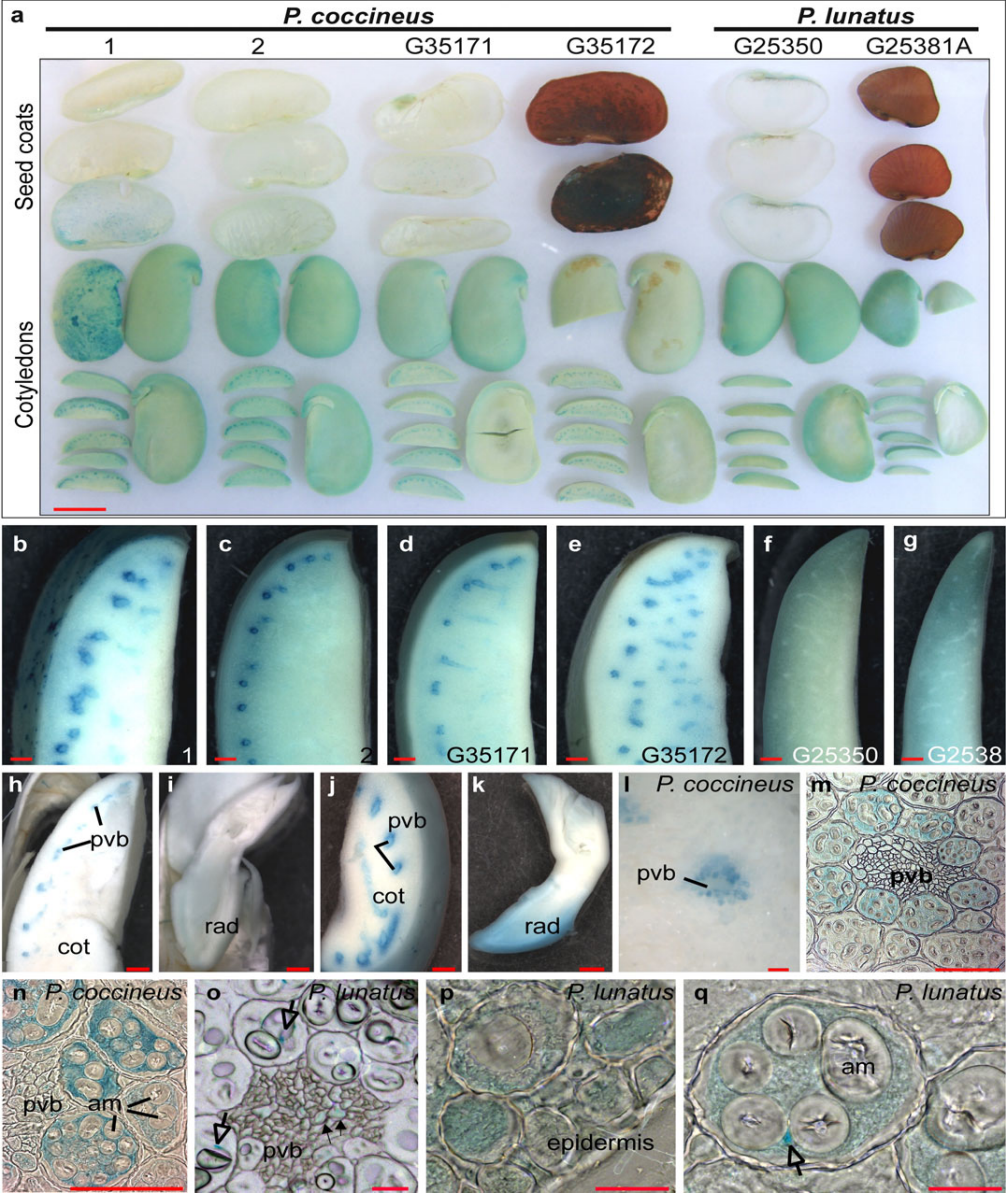
**Iron distribution in *P. coccineus *and *P. lunatus *seeds**. Perls' Prussian blue staining of *P. coccineus *and *P. lunatus *seeds. **a **to **g: **Beans soaked 18 hours prior to staining. **a: **Stained seed coats and cotyledons from two *P. coccineus *batches purchased in Denmark (1 and 2), two *P. coccineus *genotypes (G35171 and G35172), and two *P. lunatus *genotypes (G25350 and G25381A). **b **to **g: **Close up stereomicroscope pictures of the samples in **a**. **h **to **n**: *P. coccineus *batch 1, **o**: *P. lunatus *G25381, and **p**, **q**: *P. lunatus *G25350. Stereomicroscopy of stained non-soaked beans (**h **and **i**) and of beans soaked in water for 2.5 hours prior to staining (**j **and **k**). **h **and **j: **Pictures of cotyledons, the blue stained provascular region (pvb) is indicated. **i **and **k: **Images of embryonic axes. **l: **Close up picture of the provascular region. **m **to **q **are light microscopy of thin cotyledon sections. **m, n, p**, and **q: **seeds were soaked in water for 18-24 hours before dissection and staining. **o: **dry seeds were dissected and soaked in 70% ethanol for 24 hours prior to PPB staining. Filled arrows point at iron stained cells and open arrows point at small iron stained spots. am: amyloplasts, rad: radicles, pvb: provascular bundles, cot: cotyledons. Scale bars in **a**: 1 cm, in **b **to **k**: 1 mm, in **l **to **n: **0.1 mm, and in **o **to **q**: 0.01 mm.

To determine whether iron storage in the provascular region was a general property of beans from the *Phaseolus *genus, we used the PPB method to study iron distribution in *P. coccineus *and *P. lunatus *seeds. While iron is accumulated in the provascular region of the cotyledons of *P. coccineus *(Fig. 5a to 5e) and *P. vulgaris *(Fig. 2), it was not observed in this region in *P. lunatus *(Fig. 5f and 5g). Instead, the *P. lunatus *cotyledons showed a gradient of increasing iron accumulation toward the outer epidermal layers, with provascular strands visible as unstained white lines (Fig. 5f and 5g). These results indicate that the strategies for storing seed iron are specific for each species.

We studied whether soaking affected seed iron distribution. Our results show that soaking *P. coccineus *beans in water for just 2.5 hours had a significant effect on iron staining (Fig. 5h and 5i compared to 5j and 5k). Soaked tissue showed stronger iron stain compared to un-soaked tissue. It is not clear whether this is a result of increased penetration of the PPB solution into the soaked tissues or a consequence of iron mobilization. Iron was detected in the provascular region of soaked and un-soaked cotyledons, but in particular the radicles of soaked beans showed significantly stronger staining (Fig. 5k).

In agreement with the results from *P. vulgaris*, microscopical analyses of *P. coccineus *cotyledons show the accumulation of iron in the cytoplasm of the cells that surround the provascular bundles (Fig. 5m and 5n). In sections of *P. lunatus *cotyledons, iron was only detected in epidermal cells (Fig. 5p), in cells proximal to the epidermal layer (Fig. 5p and 5q), and in some cells of the provascular bundles (Fig. 5o), but not in the cells surrounding this tissue. With the exception of small iron staining spots that were found throughout the sections of cotyledons (indicated by open arrows in Fig. 5o and 5q), the iron concentration in the examined tissue was below the detection limit for the method used. Similar to *P. vulgaris *and *P. coccineus*, the iron detected in the epidermal region was found in the cytoplasm. The iron in the provascular tissue of *P. lunatus *cotyledons was detected when the dry *P. lunatus *seeds were dissected and incubated in 70% ethanol prior to staining for 35 min, but not when the seeds were soaked in water for 18 to 24 hours before staining. As in *P. vulgaris*, *P. coccineus *and *P. lunatus *amyloplasts remain unstained (Fig. 5n and 5q).

### Ferritin accumulates in the amyloplasts of mature *P. coccineus *seeds

**Figure 6:**
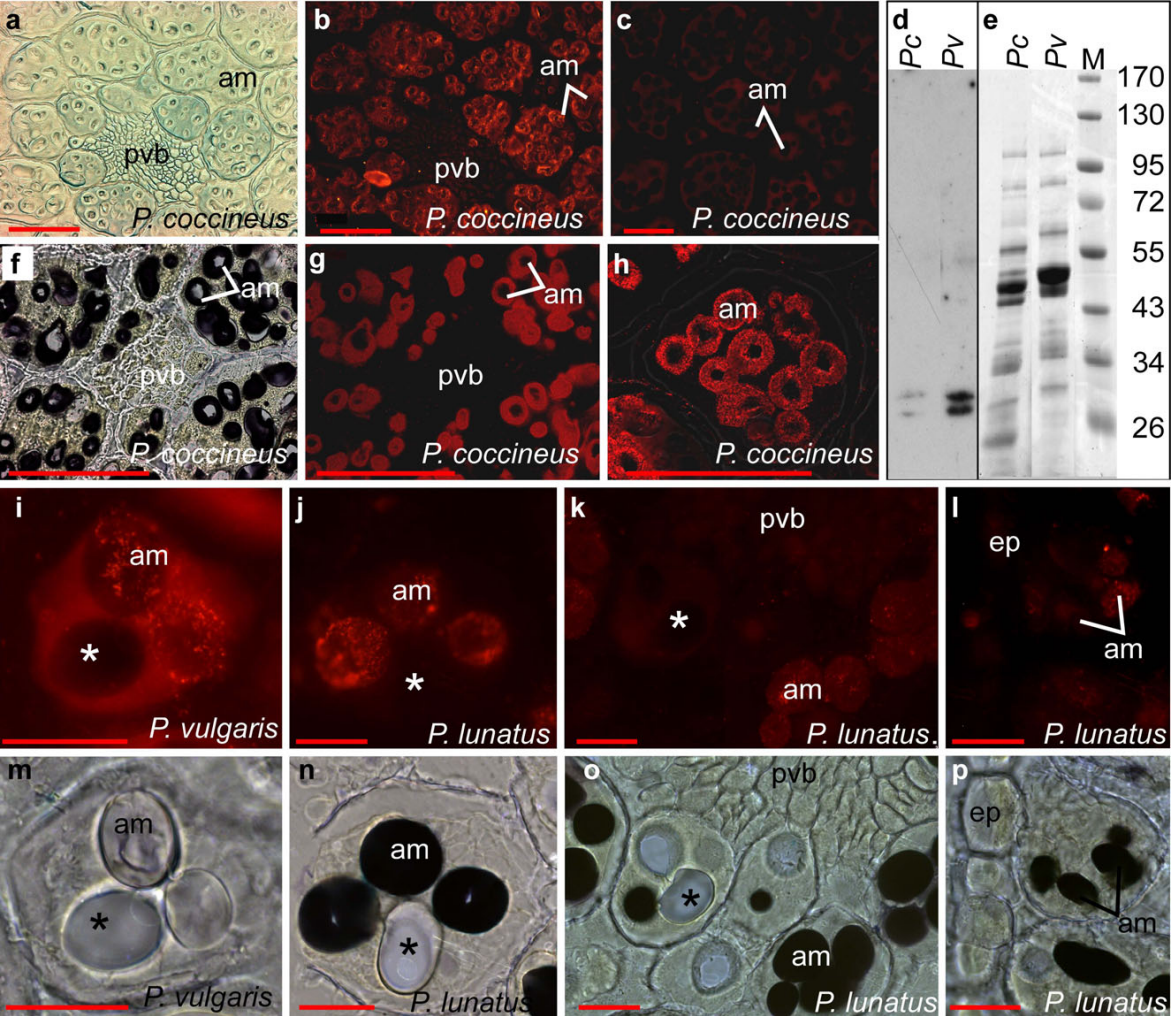
**Ferritin immunolocalization**. Sections of *P. coccineus *(**a, b, **and **f **to **h)**, *P. vulgaris *G14519 (**i **and **m**), and *P. lunatus *G25350 (**j, k, l, n, o**, and **p**) cotyledons were immunostained using antibodies raised against *A. thaliana *ferritin1 (*At*FER1) and Alexa 546 secondary antibodies. In **a, b, c, f, g, h, k, l, o, **and **p **the seeds were soaked in water for 18-24 hours prior to PPB staining, while in **i, j, m, **and **n **dry seeds were dissected and soaked in 70% ethanol for 24 hours prior to PPB staining. **b, g, i, j, k**, and **l**: fluorescence microscopy of the immunostained tissue sections shown in **a, f, m, n, o**, and **p **respectively. **a **and **m**: Light microscopy of Perls' Prussian blue stained tissue after immunostaining. In **f, n, o, **and **p **the sections were treated with Lugol solution after immunostaining; the starch is stained black. **c**: Section treated like **b, g**, and **h**, but without primary antibody. **h**: Confocal microscopy of a single immunostained cell of *P. coccineus *cotyledon. **d**: Western blot analysis of the gel shown in **e **using anti-*At*FER1 serum. **e**: Coomassie blue stained gel of 6 μg total protein from beans of *P. coccineus *(*Pc*) and *P. vulgaris *(*Pv*). **M**: Protein ladder, pvb: provascular bundle, am: amyloplasts, ep: epidermis, *: site where starch crystals were removed during sectioning. Scale bars in **a **to **c **and **f **to **h**: 0.1 mm, and in **i **to **p**: 0.01 mm

Ferritin has been suggested as the major iron-storing protein in legume seeds [16]. Therefore we decided to study whether the iron-rich cells of beans also accumulated more ferritin than the surrounding cells. Ferritin was detected in tissue sections using immunolocalization (Fig. 6). The primary antibodies used were raised against the *A. thaliana *ferritin1 [26]. Therefore we analyzed whether these antibodies were able to detect ferritin in common beans. Western blot analysis shows that the antibodies detected two bands at approximately 28 kDa in total-protein extracts from seeds of *P. coccineus *(*Pc*) and *P. vulgaris *(*Pv*) (Fig. 6d and 6e). The presence of two bands is consistent with previously published results for other plants [27]. Fluorescence microscopy of the immunostained tissue from *P. coccineus, P. vulgaris*, and *P. lunatus *cotyledons shows that ferritin accumulates in amyloplasts (Fig. 6). Sections that had been stained for iron using the PPB method were used for the immunodetection of ferritin. The pictures illustrate that ferritin does not accumulate in the cellular compartments where the iron concentrations are highest. Ferritin was only detected in the amyloplasts where the concentrations of iron were below the levels of detection for the PPB method (Fig. 4, 5, and 6). For example, iron was detected in and near the epidermal cells of *P. lunatus*, but ferritin was not detected in these cells (Fig. 5p and 6l). In the same species, iron staining was observed in the provascular cells while ferritin signals were only visible in the amyloplasts of the surrounding cells (Fig. 5o and 6k). A control sample where the primary antibody was omitted is shown in Fig. 6c. No antibody signals were observed in the amyloplasts of control sections, even when longer exposure times were used. Staining of the sections using the Lugol solution confirmed that ferritin was only detected where starch (black) was present. For example, ferritin was not detected in the regions were the starch crystals were removed during sectioning (indicated by "*" in Fig. 6i to 6k and 6m to 6o).

These results indicate that the highest iron concentrations are found in epidermal cells, in cells proximal to the epidermal layer, in provascular cells, or in cells surrounding the provascular bundles of mature *Phaseolus *seeds. Iron was primarily detected in the cytoplasm of these cells while ferritin was always detected in the amyloplasts. Although iron is likely present in ferritin, there were no correlation between the detection of iron using the PPB method and the presence of ferritin.

### High concentrations of phosphorus accumulate in the radicles and near the provascular bundles of *P. coccineus *and *P. vulgaris *beans

We used Micro-PIXE analysis to study whether there was a correlation between the accumulation of phosphorus and iron. Color maps showing the distribution of these elements in *P. coccineus *and *P. vulgaris *seeds are shown in Fig. 1. The highest phosphorus concentrations were measured in radicle tissues (Fig. 1q, t, l, o and Table 2, regions 10 to 13, 22, 24 to 26). The regions of the radicle that accumulated the most iron also had a higher concentration of phosphorus (regions 11, 12, 25, and 26). A similar pattern was observed in the regions near the provascular bundles of the cotyledons (regions 3,4,6,16,17,18, and 20). However, regions with similar phosphorus concentrations had significantly different iron content (for example region 3 and 24 compared to 5 and 25, respectively). Furthermore, low phosphorus concentrations were observed in iron-rich seed coats (region 9) and in the region near the epidermal surfaces that accumulated 80 μg g^-1 ^of iron (region 8). In the present study we were not able to determine whether iron and phosphorus co-localized at the sub-cellular level.

## Discussion

### Cells surrounding the provascular bundles can store high concentration of iron

We have discovered that in the cotyledons, the first layers of cells surrounding the provascular bundles can accumulate up to 500 μg g^-1 ^of iron in *P. vulgaris *and 300 μg g^-1 ^in *P. coccineus *genotypes (Fig. 1 and table 2). In comparison, iron was shown to accumulate in the provascular tissue of the seeds of *A. thaliana *[21]. The same study showed that the disruption of the vacuolar iron uptake transporter (VIT1) altered the distribution, but not the total concentration, of iron in seeds. It was concluded that the accumulation of iron in vacuoles is critical for seed development. Our microscopical analysis indicates that high concentrations of iron accumulate in the cytoplasm of cells surrounding the provascular bundles of mature *P. vulgaris *and *P. coccineus *seeds (Fig. 4 and 5). Our study does not reveal the cellular distribution of iron in the provascular tissue. Therefore it is possible that vacuoles in the provascular tissue are important for iron storage or for loading iron into the seeds. It is also possible that vacuolar iron is primarily needed during the early stages of germination and that the iron stored in the cytoplasm of the cells surrounding the provascular tissue represents an iron reserve that could be mobilized later during germination.

### The distribution of iron in bean seeds is dependent on species and genotype

We show that six of the seven studied genotypes of *P. vulgaris *accumulate high concentrations of iron in cells surrounding the provascular bundles of the embryonic cotyledons (Fig. 2). The distribution of iron in the seeds of four *P. coccineus *genotypes was similar to that in *P. vulgaris*, while iron was detected throughout the cotyledons of the two *P. lunatus *genotypes, which showed no increased accumulation in the cells surrounding the provascular bundles (Fig. 5).

Our analysis shows that the distribution of iron between the seed coat and embryo varies between genotypes within the same species (Table 1 and Fig. 2). Similarly, Ariza-Nieto *et al *[4] have shown that in common beans the percentage of iron accumulated in the embryo is genotype-dependent. They found that seed coat iron represented 4 to 26% of the seed iron, depending on the genotype. In comparison our estimates show that 2 to 18% of the seed iron is present in the seed coats (Table 1). A comparison of the two studies indicates that iron concentrations in seed coats and the embryonic axis are slightly higher in the study by Ariza-Nieto *et al*, even when the total seed iron concentrations were similar. These differences could be caused by iron mobilization during soaking, as the beans used by Ariza-Nieto *et al *were soaked prior to dissection. Another possibility is that the endospermal layer, which we have shown can accumulate 270 μg g^-1 ^of iron, was still attached to the seed coat of their soaked beans, while this layer was mostly removed for our analysis. Other studies of common beans have shown that 5 to 40% of the seed iron was found in the seed coats [23,28]. These are significantly higher values than found in the present study. It is possible that the genotypes studied by Moraghan *et al *(2002) accumulated more iron than the ones used in our investigation. Interestingly, Moraghan *et al *(2002) analyzed seeds that were harvested at the R7 stage of growth. It is possible that iron is mobilized to the embryo during the last stage of maturation, changing the proportion of seed coat iron between the R7 stage and maturity. In agreement with that theory, it has been suggested that iron in pea seeds could temporarily accumulate in non-vascular cells of the seed coats, thereafter mobilizing to the embryo apoplast [16].

Among the common bean genotypes analyzed here, three (CAL96, DOR364, and G19833) were in common with those of Ariza-Nieto *et al*., while the others represented contrasting genotypes selected for their high (G14519, G21242, NUA35) and low (G4825) seed iron content. These previous authors also found variability in seed coat iron with DOR364 (Mesoamerican) also having high seed coat iron relative to CAL96 and G19833 (Andeans) suggesting that gene pool differences affect this trait. Therefore, our results emphasize the importance of complementing genetic analysis with physiological analysis of the gene pools, species, and genotypes of interest. They also highlight the importance of studying iron metabolism in both model and crop plants.

Indeed, a comparison of the iron accumulation patterns of NUA35 with its parents CAL96 and G14519 suggests that iron distribution criteria should be integrated into selection strategies for bean biofortification.

### Ferritin accumulates in the amyloplasts of embryonic cells

It has been suggested that phytoferritin-bound iron is the principal iron form during early germination [15]. In peas, ferritin was shown to accumulate in the embryo and it was suggested that ferritin-iron accounted for 92% of the iron in this tissue [16]. Recent analyses of legume ferritins, which included soybeans, common beans, and peas, suggested that ferritin iron can maximally account for 18 to 42% of the total seed iron depending on the species [29]. For white and red kidney beans it was calculated that 20 and 25% of the total seed iron was bound to ferritin respectively [29]. Furthermore, it was estimated that up to 5% of the seed iron in *A. thaliana *could be bound to ferritin [30]. However, similar to the findings of Hoppler *et al*. [29] and Ravet *et al*. [30], our results indicate the accumulation of non-ferritin iron in the seeds of the three studied *Phaseolus *species. Although it is likely that ferritin contains iron, the concentrations of iron accumulated in the ferritin-rich amyloplasts was below the level of detection of the method used (Fig. 6). In contrast, our microscopical analysis indicates that the cytoplasm of cells surrounding the provascular tissue plays a key role in iron storage in the bean seeds of the *P. vulgaris *and *P. coccineus *species. Iron rich cells were also detected at and near the epidermal layer in the three studied *Phaseolus *species. In these cells, iron staining was also visible in the cytoplasm.

The accumulation of ferritin in amyloplasts is in agreement with the general knowledge that ferritins are localized in plastids [17,31,32]. A correlation between starch and ferritin accumulation in legumes is suggested by their co-localization in amyloplasts and by the knowledge that they both are degraded during seed germination [33]. Ferritin was also one of the 289 proteins found in the soluble fraction of amyloplasts from wheat endosperm [34]. The primary role of ferritin in *A. thaliana *was suggested to be the protection against reactive oxygen species [30].

Using Western blot analysis, we detected two ferritin bands in extracts from *P. coccineus *and *P. vulgaris *seeds (Fig. 6). This is in agreement with previous studies of other seeds. Two ferritin peptides of 28 kDa and 26.5 kDa were detected in extracts from soaked pea, soybeans, and maize seeds [35]. The products of at least two ferritin genes were identified in soybean seeds, while only one of the ferritins from *A. thaliana*, AtFER2, was detected in mature seeds [27,36,30].

### Seed regions near the provascular bundles are rich in iron and phosphorus

The accumulation of ferritin has recently been shown to be affected both by the iron and the phosphate status of *A. thaliana *plants [37]. Furthermore, the study showed that the accumulation of iron in leaves was shifted from vacuoles to chloroplasts under phosphate deficiency. It was suggested that the vacuolar iron was found in phosphorus complexes that were formed when plants were grown in phosphate-rich medium, while chloroplast iron was bound to ferritin [37]. In this study the distribution of phosphorus was visualized using micro-PIXE analysis (Fig. 1). The iron-rich regions near the provascular bundles of the cotyledons and of the radicle accumulated a higher proportion of the seed phosphorus compared to the surrounding regions. In contrast, iron-rich regions of the seed coat and near the epidermal cells did not accumulate considerable amounts of phosphorus.

Therefore, it is possible that some percentage of the seed iron can be bound to phosphorus-rich compounds, but we suggest that the cells near the provascular tissues have a general role in nutrient storage that explains the higher concentration of phosphorus. For example iron and manganese accumulate near the provascular bundles of *A. thaliana *seeds [21]. Their distribution patterns differ slightly, indicating that they are not part of common complexes and emphasizing the role of the region near the provascular bundles in the storage of nutrients. Similarly, we suggest that the radicle might have an important function in storing nutrients that are needed during the early events of germination.

The iron-rich cells of the provascular region of the *Phaseolus *species show similarities to the extended cells surrounding the provascular bundles of the cotyledons and leaves of the non-legume *Ricinus communis *(castor bean). These cells were shown to accumulate the iron chelator nicotianamine (NA) [38]. In strategy I plants, NA is suggested to be involved in binding ferrous iron in the phloem sap [39] and was shown to be important for the supply of iron to seeds in *A. thaliana *[22]. Our PPB staining indicates that the iron accumulated near the provascular bundles of beans is primarily ferric iron. NA can bind both ferrous and ferric iron with similar affinities [40,41]. Therefore it is possible that NA is involved in chelating iron in the iron-rich cells of the provascular region in beans.

Taken together, we show that the distribution of iron in seeds depends on the species and genotype and that the cells surrounding the provascular tissues of the embryo might play a key role in the storage of minerals in mature seeds. Furthermore, our results indicate that a large proportion of the seed iron in the *Phaseolus *species is stored in compounds different from ferritin and that accumulation of iron in the seed coat is highly variable between *P. vulgaris *genotypes. In addition, we suggest that the PPB technique is ideal to study iron distribution in legume seeds during plant breeding and to detect seed-to-seed variations. This technique may contribute to a quick and inexpensive method for the selection of genotypes with more bioavailable iron.

Future studies of the molecular mechanisms behind the described accumulation of nutrients and their importance for seed germination will shed new light on how seeds store iron and on the possibility to improve the nutritional value of seeds.

## Conclusions

Using two different techniques we were able to show that cells surrounding the provascular tissue contain a high concentration of iron in *P. coccineus *and *P. vulgaris *seeds. This cell layer appears to be of key importance for iron storage in the seed or for providing iron to the germinating seedling. Therefore these cells are an appropriate target for future molecular biology research.

In agreement with previous studies in legumes [29] and in *A. thaliana *[30] our studies indicate that high concentrations of non-ferritin-iron is accumulated in the seeds of *P. coccineus, P. lunatus*, and *P. vulgaris*. These findings emphasize the need to characterize other compounds that might be involved in the chelation of iron in mature seeds and the proportion of iron bound by the different chelators as well as investigating the bioavailability of iron from different sources.

Recent research in *A. thaliana *indicates that vacuolar transporters are important for seed iron distribution and for iron loading to the seedling during germination [20,21]. Our findings suggest that high concentration of iron is accumulated in the cytoplasm of cells surrounding the provascular tissue of the *P. coccineus *and *P. vulgaris *seeds. These results indicate either that there are major differences between *A. thaliana *and the *Phaseolus *species with respect to the subcellular localization of iron accumulation, or that the vacuolar transporters are one of several transporters involved in iron mobilization to and from the seeds. In the latter case, additional transporters might be involved in the loading of iron to the cytoplasm of iron-rich cells that surround the provascular tissue of some legume species.

## Methods

### Plant materials

*Phaseolus vulgaris *(7), *P. coccineus *(2) and *P. lunatus *(2) genotypes were obtained from the International Center for Tropical Agriculture, Cali, Colombia and are maintained either with the Genetic Resource Unit (G entries) or with the Bean Program (CAL, DOR and NUA lines) at CIAT. All *P. vulgaris *genotypes were grown both in Darien, Valle de Cauca, Colombia and in a greenhouse in Denmark. Darien is 1400 m above sea level, at this location the growth conditions were: 20°C average yearly temperature, 1288 mm annual rainfall, Udand soil type, pH 5.6, and native HCl extractable mineral concentration for iron in the topsoil averaged 4.39 μg g^-1^. In the greenhouse, plants were grown in soil in five liter pots, 18-23°C daytime/15-20°C nighttime temperatures, 16 hours light/8 hours dark cycle, and 70% relative humidity. The plants were watered with a 0.1% solution of Pioneer NPK (14-3-23) + Mg (Blue) (The Broste Group - Ltd. Reg. No. 39781 http://www.broste.com), 50% additional S was added as NH_4_SO_4_. If not otherwise stated, the results shown are from seeds grown for one season in Darien. In the Danish greenhouse, the genotypes were grown twice and seeds were harvested in August-September of both 2008 and 2009.

Two batches of *P. coccineus *beans were purchased at a retailer in Denmark. The *P. vulgaris *accessions and breeding lines are from both the Mesoamerican (small-seeded) and Andean (large-seeded) gene pools of common bean as indicated in Table 1.

### Iron quantification

For each genotype, ten to fifteen dry mature seeds (depending on seed size) were dissected into embryo axis, cotyledons, and seed coat tissues using a razor blade. The endospermal layer between the cotyledons and the seed coats was collected separately in order to avoid contamination between these two tissues. The iron content of each tissue type was measured in duplicates at the Danish Technological Institute, Kongsvang Alle 29, Aarhus, Denmark, using inductively coupled plasma atomic emission spectroscopy (ICP-AES) in axial mode.

### Iron biochemical stains

Perl's Prussian blue (PPB) method was used as previously described [42,43]. In short, 1 volume of 4% HCl solution was mixed with 1 volume of a fresh 4% solution of Potassium hexacyanoferrate (II) trihydrate and used to cover the tissue. Some beans were soaked for 2.5, 18, or 24 hours in water at 20°C, sliced, and incubated in 70% ethanol for 1-2 hours, others were sliced without prior soaking. The tissue was covered with the solution and allowed to react for 15 to 35 minutes before washing in pure water at least 5 times. Thereafter the tissue was kept in 70% ethanol in the dark until analysis.

### Preparation of samples for light microscopy

Samples that were stained with PPB method as previously described were incubated 2 to 5 days in 70% ethanol prior to fixation. The tissue was thereafter fixed for 24 to 48 hours at 4°C in 4% paraformaldehyde, 1% glutaraldehyde, 0.1 M NaHPO_4 _pH 7.2. A stepwise dehydration in ethanol was performed using 30 minutes incubations in 70%, 80%, 90%, and twice in 96% ethanol. For embedding and polymerization we used the Technovit 7100 kit (Heraeus Kulzer, Wehrheim, Germany). Embedding was performed using stepwise increments of Technovit hardener I in ethanol, and manufacturers' recommendations were followed for the polymerization. The specimens were sectioned into 7 to 11 micrometer thick samples using a Leica RM2045 microtome and studied by light microscopy.

### Immunoassays

Glass slides with sections of PPB stained and unstained tissue, fixed as previously described, were blocked for an hour at room temperature (RT) using PBS-blotto solution (20 mM phosphate pH 7.4, 150 mM NaCl, 0.1% (v/v) triton X-100, 3% (w/v) nonfat dry milk). Thereafter the sections were incubated with rabbit serum raised against *A. thaliana *ferritin 1 [26], diluted 2000 times in PBS-blotto. For control samples PBS-blotto without serum was used. The incubation proceeded for 12 to 18 hours at 4°C. The slides were rinsed twice in PBS-T (20 mM phosphate pH 7.4, 150 mM NaCl, 0.1% (v/v) triton X-100), and washed 3 times for 5 minutes at room temperature in PBS-T. Thereafter the slides were incubated with Alexa fluor 546 goat anti-rabbit secondary antibodies (Invitrogen), which was diluted 400 times in PBS-blotto, for 1 to 3 hours at RT. The slides were rinsed twice with PBS-T, washed 3 times for 5 minutes in PBS-T, fixed for 10 minutes in 2% paraformaldehyde in PBS (20 mM phosphate buffer pH 7.4, 150 mM NaCl), and washed 3 times in PBS-T. Microscopy was performed using Zeiss Axioplan fluorescence microscope and Zeiss LSM510 Meta confocal microscope (excitation 543 nm, emission filter BP 585 to 620 nm).

### Western Blots

Cotyledons were dissected from mature dry seeds of *P. coccineus *and *P. vulgaris *and ground using liquid nitrogen. Soluble proteins were extracted in 50 mM Tris-HCl pH 8.0, 2% (w/v) SDS, 1% (v/v) proteinase inhibitor cocktail (Sigma P9599). A total of 6 μg total protein was loaded in each lane. The protein was denatured in 77 mM ammediol, 0.057 N HCl, 13% glycerol, 30 mM SDS, 0.03% bromophenolblue, and 15 μM DTT for 10 minutes at 95°C. The samples and 5 μl pre-stained protein ladder (Fermentas, SM#0671) were separated in 6 to 12% polyacrylamide gradient gels as previously described [44,45]. The gels were either stained with Coomassie Brilliant Blue using standard protocols or electro-blotted to PVDF membranes. For immuno-detection the membranes were blocked using PBS-blotto as described for immunoassays, and incubated for 12 to 18 hours at 4°C with rabbit serum raised against *A. thaliana *ferritin 1 [26], diluted 10,000 times in PBS-blotto. The membranes were washed three times for 15 minutes in PBS-T, incubated 2 hours at room temperature with peroxidase-conjugated goat anti-rabbit antiserum (Dako P0448), and washed once for 15 minutes and three times for 5 minutes with PBS-T. The antibodies were detected using the ECL technique [46] and Amersham Hyperfilm™ ECL.

### Elemental distribution and quantification by micro-PIXE

Dry seeds were cut with a razor blade and mounted on a thin Formvar layer. The seed surfaces were coated with carbon. Microanalyses were performed using the nuclear microprobe at the Materials Research Department, iThemba LABS, South Africa as previously described [47-49]. In short, a proton beam of 3.0 MeV energy and 100 to 150 pA current was focused on a 3 × 3 μm^2 ^spot and raster scanned over the areas of interest, using square or rectangular scan patterns with a variable number of pixels (up to 128 × 128). Particle-induced X-ray emission (PIXE) and proton backscattering spectrometry (BS) were used simultaneously. Elemental concentrations were obtained using GeoPIXE II software [50]. Quantitative elemental images were generated using the *Dynamic Analysis *method, an integral part of GeoPIXE II. Information on atomic ratios of light elements forming organic matrix, necessary for PIXE quantitative analysis, was found from BS technique. Regions representing various seed parts were selected within seed maps on the basis of their morphology from optical micrographs and of iron distribution images. PIXE and BS spectra were extracted from these regions to obtain average concentrations within them.

## Abbreviations

ICP-AES: inductively coupled plasma atomic emission spectroscopy; NA: nicotianamine; PIXE: Particle Induced X-ray Emission; PPB: Perl's Prussian blue; ROS: reactive oxygen species; RT: room temperature.

## Authors' contributions

CC drafted the manuscript, responsible for the design and coordination of experiments, and participated in the execution of the experiments, WJP and JMP carried out the micro-PIXE analysis and helped to draft the manuscript, DFU carried out Western blots, AMJ participated in microcopy analysis, MWB and CA are responsible for breeding the high-iron bean genotypes and helped to draft the manuscript, EØJ and JS contributed to the design of experiments and helped to draft the manuscript. All the authors read and approved the final manuscript.
